# Minimally invasive extracorporeal CO_2_ removal in hypercapnic respiratory failure: a prospective observational study

**DOI:** 10.1186/s13054-026-06062-1

**Published:** 2026-04-30

**Authors:** Vitalii Kryvenko, Faeq Husain-Syed, Elisa Schnell, Gani Oruqaj, Rory E. Morty, Susanne Herold, Matthias Hecker, Khodr Tello, Werner Seeger, István Vadász

**Affiliations:** 1 Department of Internal Medicine (Pulmonology, Gastroenterology, Nephrology, Intensive Care Medicine and Palliative Medicine), Universities of Giessen and Marburg Lung Center, Justus Liebig University, Klinikstr. 33, 35392 Giessen, Germany; 2 German Center for Lung Research, Giessen, Germany; 3 Institute for Lung Health, Giessen, Germany; 4 The Cardio-Pulmonary Institute, Giessen, Germany; 5 Interdisciplinary ECMO Center, Interdisciplinary Center for Intensive Care Medicine, University Hospital Giessen, Giessen, Germany; 6 Department of Translational Pulmonology and the Translational Lung Research Center Heidelberg, Heidelberg University Hospital, Heidelberg, Germany; 7 Department of Internal Medicine (Infectious Diseases and Infection Control), Universities of Giessen and Marburg Lung Center, Justus Liebig University, Giessen, Germany; 8 German Center for Infection Research, Giessen, Germany

**Keywords:** ARDS, CO_2_, CO_2_ removal, ECCO_2_R, miECCO_2_R, CRRT, Driving pressure

## Abstract

**Background:**

Lung-protective ventilation in acute respiratory distress syndrome (ARDS) can lead to hypercapnia, an independent risk factor for increased mortality. Extracorporeal CO_2_ removal (ECCO_2_R) enables further reduction of ventilator intensity, but its routine use is limited due to safety concerns. In the current study, we evaluated the feasibility, efficacy, and safety of minimally invasive ECCO_2_R (miECCO_2_R) implemented via a renal replacement therapy (RRT) platform in patients with mild-to-moderate ARDS and refractory hypercapnia.

**Methods:**

In this prospective single-center observational study, 20 ICU patients with persistent hypercapnia despite escalated ventilation received either standalone miECCO_2_R (n = 11) or miECCO_2_R combined with continuous RRT (n = 9). As a primary outcome, efficacy of miECCO_2_R was assessed. Moreover, ventilator parameters, disease severity, renal function, and adverse events were evaluated as secondary outcome parameters over a time-course of five days upon initiation of miECCO_2_R.

**Results:**

miECCO_2_R led to a rapid and sustained reduction in PaCO_2_ levels from 71.4 mm Hg to 51.6 mm Hg within 24 h. This was accompanied by normalization of pH, and the median CO_2_ clearance rate was 64.5 mL/min. Driving pressure decreased significantly from 22 cm H_2_O to 15 cm H_2_O by day 5, while oxygenation remained stable. The standalone miECCO_2_R treatment group demonstrated faster CO_2_ reduction, probably due to higher blood flow rates. There were no severe adverse events related to either the device or the therapy. Circuit clotting was managed by system exchange, without clinical consequences for the patients. Platelet counts declined moderately, but no major bleeding complications occurred.

**Conclusions:**

miECCO_2_R delivered via an RRT platform appears to be a safe and effective method of controlling hypercapnia and facilitating lung-protective ventilation in patients with ARDS. These findings need to be supported by further randomized controlled trials that can more definitely demonstrate the impact of miECCO_2_R on clinical outcomes.

**Supplementary Information:**

The online version contains supplementary material available at 10.1186/s13054-026-06062-1.

## Background

Lung-protective mechanical ventilation is pivotal in the treatment of acute respiratory distress syndrome (ARDS) [1]. Such strategies mitigate ventilator-induced lung injury but can cause hypercapnia due to insufficient carbon dioxide (CO_2_) clearance. Elevated CO_2_ levels are an independent risk factor for increased mortality in various pulmonary diseases and in mechanically ventilated patients in intensive care unit (ICU) settings [2, 3]. Recent clinical trials show that extracorporeal CO_2_ removal (ECCO_2_R) is effective in limiting CO_2_ accumulation in patients with ARDS and maintaining lung-protective ventilation, even under ultra-protective tidal volumes [4–9]. However, severe adverse events have also been reported, some of which may be attributable to technological limitations [10]. Accordingly, the most recent European Society of Intensive Care Medicine guidelines on ARDS do not recommend routine ECCO_2_R use [1]. Instead, it is recommended that the potential benefits and harms of ECCO_2_R in this patient group will be evaluated in the setting of prospective studies [8]. In clinical practice, increasing ventilator settings, even over recommended limits, often fail to control increasing CO_2_ levels in patients with acute respiratory failure. In such cases, minimally invasive ECCO_2_R (miECCO_2_R), operated at very low blood flow rates (100–400 mL/min), similar to those employed during renal replacement therapy (RRT), may be valuable for CO_2_ removal. A previous report by the current researchers described the feasibility and safety of ECCO_2_R delivered via a RRT platform in four patients with COVID-19–associated ARDS and refractory hypercapnia, demonstrating rapid CO_2_ reduction [11]. In addition, the successful use of low blood flow rates during ultraprotective ventilation in combination with RRT was recently reported [12]. The present observational study expands on those findings through quantitative analyses of the potential of miECCO_2_R at very low blood flow rates, with or without RRT, in mechanically ventilated mild-to-moderate ARDS cases with hypercapnia.

## Methods

This prospective, single-center, observational study included 20 patients diagnosed with mild-to-moderate ARDS and persistent hypercapnia despite escalation of ventilation settings admitted to the medical ICU at the University of Giessen Lung Center between May 2020 and January 2025. Patients received miECCO_2_R as standalone therapy (n = 11; 55%) or in combination with continuous RRT (CRRT) (miECCO_2_R/CRRT) (n = 9; 45%). Initiation of CRRT was at the discretion of the treating physician and guided by Kidney Disease: Improving Global Outcomes (KDIGO) recommendations (detailed criteria are provided in eTable 1).

miECCO_2_R was performed using a polymethylpentene hollow fiber gas-exchange membrane (multiECCO_2_R; Eurosets, Medolla, Italy) and the multiFiltrate CRRT system (Fresenius Medical Care, Bad Homburg, Germany). For standalone miECCO_2_R, blood flow was set at 300–400 mL/min. For combined miECCO_2_R/CRRT, continuous veno-venous hemodialysis was applied with blood flow set to 100–200 mL/min. According to the manufacturer’s instructions, the multiECCO_2_R membrane was positioned downstream of the hemofilter (Ultraflux AV 1000S; Fresenius Medical Care, Bad Homburg, Germany) within the extracorporeal circuit, and sweep gas flow was set at approximately 15-fold the blood flow rate (4.5–6 L/min and 1.5–3 L/min for miECCO_2_R and combined miECCO_2_R/CRRT, respectively) [11]. The miECCO_2_R group received systemic anticoagulation with systemic heparin or argatroban (target aPTT 60–80 s), whereas the miECCO_2_R/CRRT group received regional citrate and prophylactic systemic anticoagulation therapy (detailed protocols are provided in the Supplementary Methods).

Demographics and baseline clinical characteristics were recorded at enrolment. The clinical variables analyzed were respiratory rate, mean arterial pressure, need for vasopressor (norepinephrine) administration, arterial partial pressure of CO_2_ (PaCO_2_), CO_2_ clearance rate, pH, bicarbonate concentration, driving pressure, arterial partial pressure of O_2_ (PaO_2_), peak inspiratory pressure (PInsp), positive end-expiratory pressure (PEEP), and fraction of inspired O_2_ (FiO_2_). Clinical severity scores and the laboratory parameters white blood cell and platelet counts, serum creatinine, urea, and hemoglobin were also analyzed. Data were collected at baseline, 1 h, 4 h, 24 h, and daily thereafter. No sample size calculation was conducted, as the study did not involve formal hypothesis testing and was intended to provide descriptive and exploratory insights only. Unless specified differently, values are presented as median (IQR 25%–75%). Repeated measures were analyzed with the Friedman, Skillings-Mack test and post-hoc Wilcoxon signed-rank test. Statistical significance was set at p < 0.05. Statistical analyses were performed using SPSS Statistics (IBM, Version 29.0.1.1) and the R software (Version 4.4.1; tidyplot package).

## Results

### Patients and disease course

The demographic and clinical characteristics of the patients and the subgroups at inclusion are presented in eTable 2 and eTable3. Although the original observation period was 72 h, the analysis was extended to 5 days based on the observed median treatment duration. By day 5, miECCO_2_R was discontinued in two patients who showed clinical improvement, while three patients died from multiorgan failure.

### Overall treatment outcomes

The mean extracorporeal blood flow rate of the miECCO_2_R system was 291.1 ± 114.8 mL/min across all patients and study visits: 380.7 ± 53.2 mL/min in the miECCO_2_R-alone group and 173.2 ± 46.8 mL/min in the miECCO_2_R/CRRT-combination group. The mean sweep gas flow rate was 4.4 ± 1.7 L/min for the overall cohort: 5.7 ± 0.8 L/min in the miECCO_2_R-alone group and 2.6 ± 0.7 L/min in the miECCO_2_R/CRRT-combination group (Table 1).

**Table 1:** Operational characteristics of miECCO_2_R

Time	Blood flow (mL/min)		Sweep gas flow (L/min)		aPTT (sec)		Post-membrane iCa^2+^ (mMol/L)	
	**miECCO**_**2**_**R** **(N = 11)**	**miECCO**_**2**_**R/CRRT** **(N = 9)**	**miECCO**_**2**_**R** **(N = 11)**	**miECCO**_**2**_**R/CRRT** **(N = 9)**	**miECCO**_**2**_**R** **(N = 11)**	**miECCO**_**2**_**R/CRRT** **(N = 9)**	**miECCO**_**2**_**R** **(N = 11)**	**miECCO**_**2**_**R/CRRT** **(N = 9)**
1 h	400 (400–400)	175 (100–200)	6.0 (6.0–6.0)	2.5 (1.5–3.0)	71 (57–88)	42 (34–46)	1.13 (1.00–1.16)	0.30 (0.26–0.34)
24 h	400 (390–400)	175 (140–200)	6.0 (6.0–6.0)	2.5 (1.9–3.0)	88 (65–116)	44 (37–48)	1.12 (1.03–1.16)	0.28 (0.25–0.31)
48 h	400 (390–400)	175 (140–200)	6.0 (6.0–6.0)	2.5 (1.9–3.0)	69 (64–82)	41 (36–48)	1.13 (1.02–1.19)	0.30 (0.28–0.31)

Data are presented as median (25%–75% interquartile range)

CRRT: continuous renal replacement therapy, miECCO_2_R: minimally invasive extracorporeal CO_2_ removal, iCa^2+^: ionized calcium

Arterial blood gas analysis revealed a rapid reduction in PaCO_2_, from 71.4 mm Hg (IQR: 64.2–77.5) at baseline to 51.6 mm Hg (IQR: 45.1–57.7) within the first 24 h of miECCO_2_R therapy, accompanied by normalization of blood pH (eTable 4). The highest CO_2_ clearance rates were observed during the first hour of miECCO_2_R treatment, with the highest value being 82.9 mL/min (IQR: 50.7–110.7). Overall, the median CO_2_ clearance across all study visits was 64.5 mL/min (IQR: 41.1–91.9).

Ventilator parameters demonstrated a significant reduction in driving pressure, with the greatest decrease observed on day 5: 22 cm H_2_O (IQR: 19–22) at baseline and 15 cm H_2_O (IQR: 13–18) on day 5. This was achieved through a gradual decrease in PInsp with maintenance of stable PEEP (eTable 5). Analysis of lung mechanics revealed an increase in dynamic respiratory compliance and a reduction in mechanical power and ventilatory ratio over the observation time-course. The reduction in PaCO_2_ was achieved under preservation of arterial oxygenation. While PaO_2_ remained stable, the PaO_2_/FiO_2_ ratio increased to 185.0 (IQR: 148.9–252.5) by day 5. These improvements were accompanied by a reduction in vasopressor requirements and maintenance of a mean arterial pressure above 65 mmHg.

No significant changes were observed in clinical severity scores, including SOFA, APACHE II, and SAPS II, throughout the study period. Platelet counts significantly reduced from 263 × 10⁹/L (IQR: 211–344) at baseline to 198 × 10⁹/L (116–256) at day 3.

### Standalone miECCO_2_R versus combined miECCO_2_R/CRRT

**Fig. 1:**
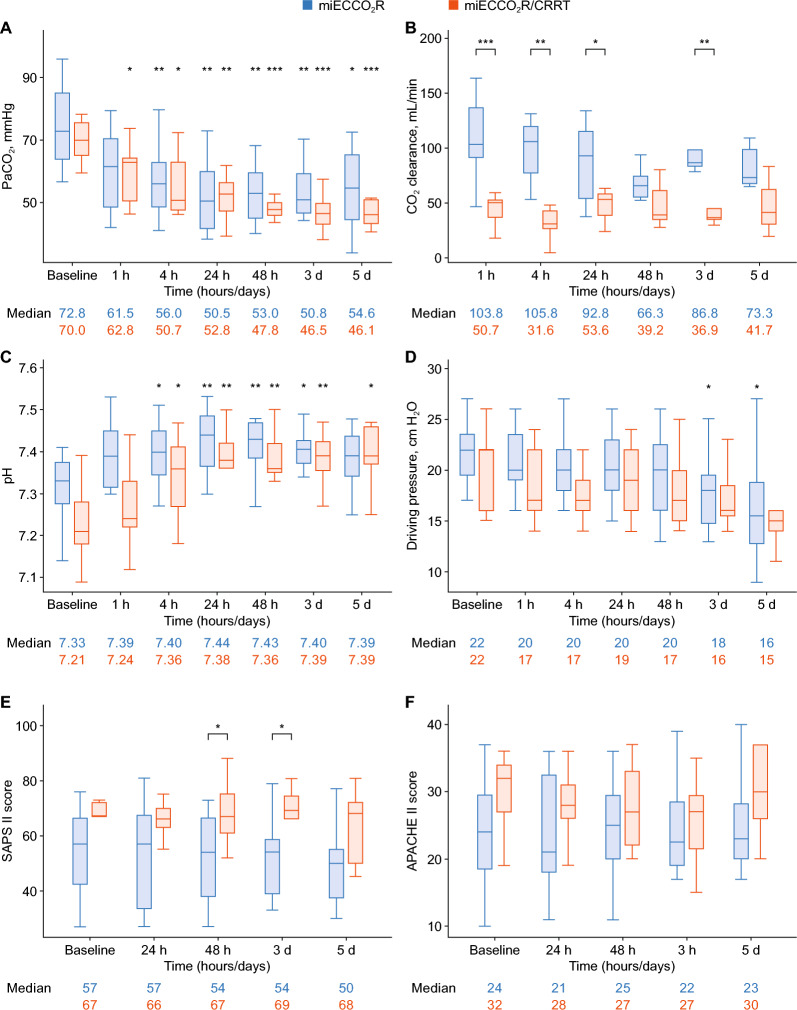
Key treatment outcomes of miECCO_2_R with and without CRRT. The graphs depict the course of PaCO_2_ (**A**), CO_2_ clearance (**B**), pH (**C**), driving pressure (**D**), and clinical scores (**E** and **F**) in patients with mild-to-moderate ARDS and refractory hypercapnia receiving either miECCO_2_R as standalone therapy (blue) or in combination with CRRT (red). The boxplots display median values with 25%–75% interquartile ranges as well as minimum and maximum values at baseline and up to 5 days upon initiation of miECCO_**2**_R. *p < 0.05, **p < 0.01, ***p < 0.001, compared to baseline (**A**, **C**, and **D**) or miECCO_**2**_R compared to miECCO_**2**_R/CRRT (**B** and **E**)

Compared to combined miECCO_2_R/CRRT, standalone miECCO_2_R resulted in significantly faster PaCO_2_ reduction, normalization of pH, greater CO_2_ clearance, and decreased driving pressure, particularly within the first 24 h (Fig. 1). This probably reflects the higher blood flow rates, one of the major determinants of CO_2_ removal efficacy, with standalone miECCO_2_R, that enabled greater CO_2_ elimination and may also be influenced by greater illness severity in the combined treatment group, as indicated by higher SAPS II and APACHE II scores. Serum creatinine remained largely stable over time in the standalone miECCO_2_R group. As expected, the combination group demonstrated a significant improvement in serum creatinine.

### Treatment-related adverse events

No severe adverse events related to miECCO_2_R therapy were observed in either group. In one patient, a single bleeding episode occurred at the tracheostomy site, but platelet counts and coagulation parameters were within the normal or predefined target range at the time of the event. In another case, escalation to extracorporeal membrane oxygenation was required due to rapid clinical deterioration with hypoxemia. This was considered unrelated to miECCO_2_R therapy and not classified as a treatment-associated severe adverse event. Clot formation within the extracorporeal circuit occurred in 4 cases (for additional information please refer to the Supplementary Appendix). When considering the total number of circuits used, the effective clotting rate per circuit was 7.7% and therefore within the range reported for CRRT systems, where circuit lifespan is typically limited to approximately 26–47 h depending on the anticoagulation strategy, including a median of ~ 47 h with regional citrate anticoagulation in CRRT [13]. Similar ranges have been reported in a recent study using CRRT combined with ECCO_2_R [12]. In each instance, clotting was successfully resolved by changing the CO_2_ filter or the miECCO_2_R circuit, without further clinical consequences.

## Discussion

The present findings suggest that miECCO_2_R is a safe and effective addition to mechanical ventilation for patients with mild-to-moderate ARDS and refractory hypercapnia. CO_2_ levels and ventilator pressures were significantly reduced without any compromise in oxygenation and hemodynamic stability or occurrence of major complications. Implementation of miECCO_2_R via an RRT platform was feasible, particularly in cases with indications for RRT.

Due to the high CO_2_ elimination rate despite ultra-low blood flow levels, normocapnia could be achieved in most patients within 24 h. This allowed for subsequent de-escalation of ventilation. This is of particular relevance, as it is known that both hypercapnia and injurious ventilation are independent risk factors of mortality in ICU patients requiring respiratory support [3, 14, 15].

The design of our study differs from previously published trials, in which the primary goal was to lower tidal volume while maintaining normal or slightly elevated PaCO_2_ with ECCO_2_R [4, 5, 16]. In our severely hypercapnic cohort, tidal volume was ~ 6 mL/kg; however, the driving pressure was well above the recommended levels at initiation of miECCO_2_R. Our primary objective was, therefore, to both reduce the markedly elevated PaCO_2_ levels and de-escalate ventilator pressures, thereby lowering the risk of barotrauma while preserving gas exchange. We were able to achieve both goals with standalone and combined treatments and, thereby, demonstrate the feasibility and benefits of miECCO_2_R by itself and when combined with RRT.

Patients in our study exhibited marked disease severity, with baseline SAPS II, SOFA, and APACHE II scores corresponding to an expected hospital mortality of approximately 50–70% [17–19]. In our collective, we observed a 45% 28-day mortality in patients receiving miECCO_2_R therapy with or without RRT. While our study was neither designed nor powered to detect a mortality benefit with miECCO_2_R, it is conceivable that achieving normocapnia and limiting barotrauma with a minimally invasive method and a potentially low incidence of adverse effects may lead to better outcomes in this patient group.

Major bleeding is a severe adverse event associated with ECCO_2_R [4, 10]. However, in our cohort, no major bleeding events were recorded. The use of an RRT platform as a “motor” of miECCO_2_R (a peristaltic pump) may be favorable in the context of hemolysis, as the centrifugal pumps regularly used for ECCO_2_R are associated with high rates of hemolysis at low blood flow levels [10].

While the present findings demonstrate the benefits of miECCO_2_R in the examined patient group, certain limitations need to be considered, such as the small sample size, the single-center design, the absence of a control group and the lack of standardized ventilatory management due to the explorative nature of the study. Also, the observational period was limited to five days after initiation of miECCO_2_R as at later time-points meaningful comparisons would have been progressively more difficult due to the increasing amount of missing data (smaller sample size over time). Moreover, patients requiring CRRT had an independent indication for renal support and may therefore represent a subgroup with more advanced organ dysfunction, as reflected by higher baseline SOFA scores, introducing potential selection bias. Importantly, in the group of combined miECCO_2_R and CRRT extracorporeal blood flow rates were significantly lower, whereas the rate of ARDS secondary to COVID-19 was higher, when compared to standalone miECCO_2_R; accordingly, comparisons between groups should be interpreted with caution.

## Conclusion

The delivery of miECCO_2_R via an RRT platform presents a scalable, resource-efficient strategy in mechanically ventilated patients with mild-to-moderate ARDS. A multicenter prospective randomized controlled trial is necessary to demonstrate the effect of miECCO_2_R on clinical outcomes in this patient group.

## Supplementary Information

Additional file 1.

## Data Availability

The datasets used and analyzed are during the current study are available from the corresponding author on reasonable request.

## Abbreviations

APACHE II: Acute physiology and chronic health evaluation score II
ARDS: Acute respiratory distress syndrome
CRRT: Continuous renal replacement therapy
ICU: Intensive care unit
miECCO_2_R: Minimally invasive extracorporeal carbon dioxide removal
PaCO_2_: Arterial partial pressure of carbon dioxide
PaO_2_: Arterial partial pressure of oxygen
PEEP: Positive end-expiratory pressure
PInsp: Peak inspiratory pressure
RRT: Renal replacement therapy
SAPS II: Simplified acute physiology score II
SOFA: Sequential organ failure assessment score
