# Supplementary material for: Minimally invasive extracorporeal CO2 removal in hypercapnic respiratory failure: a prospective observational study
Source: Crit Care. 2026 Apr 30;30:224. doi: 10.1186/s13054-026-06062-1 (PMC13130721; doi:10.1186/s13054-026-06062-1)
Supplement: Supplementary file 1 — Additional file 1. [file 13054_2026_6062_MOESM1_ESM.docx]

**SUPPLEMENTARY APPENDIX**

**Minimally invasive extracorporeal CO_2_ removal in hypercapnic respiratory failure: A prospective observational study**

Vitalii Kryvenko^1,2,3,4*^, Faeq Husain-Syed^1,2*^, Elisa Schnell^1,2^, Gani Oruqaj^1,2,5^, Rory E. Morty^2,6^, Susanne Herold^2,3,4,7,8^, Matthias Hecker^1,2,5^, Khodr Tello^1,2,3,4,5^, Werner Seeger^1,2,3,4^, István Vadász^1,2,3,4,5^

*^1^Department of Internal Medicine (Pulmonology, Gastroenterology, Nephrology, Intensive Care Medicine and Palliative Medicine), Justus Liebig University, Universities of Giessen and Marburg Lung Center, Giessen, Germany*

*^2^German Center for Lung Research, Giessen, Germany*

*^3^Institute for Lung Health, Giessen, Germany*

*^4^The Cardio-Pulmonary Institute, Giessen, Germany*

*^5^Interdisciplinary ECMO Center, Interdisciplinary Center for Intensive Care Medicine, University Hospital Giessen, Giessen, Germany*

*^6^Department of Translational Pulmonology and the Translational Lung Research Center Heidelberg, Heidelberg University Hospital, Heidelberg, Germany*

*^7^Department of Internal Medicine (Infectious Diseases and Infection Control), Justus Liebig University, Universities of Giessen and Marburg Lung Center, Giessen, Germany*

*^8^German Center for Infection Research, Giessen, Germany*

*^*^These authors contributed equally to this work and share first authorship*

This supplementary material has been provided by the authors to give readers additional information about their work.

**Supplemental Methods**

**ECCO_2_R system management and exchange**

Blood flow and sweep gas flow were adjusted in accordance with the manufacturer’s recommendations for the multiFiltrate system in combination with the multiECCO_2_R filter. Sweep gas flow was regulated proportionally to blood flow (maximum sweep gas flow = 15 × blood flow) following the instructions of the manufacturer and also based on previous reports documenting maximal efficacy of low flow ECCO_2_R systems at a 15:1 ratio (1, 2), as summarized below.

| **Blood flow (mL/min)** | **Sweep gas flow (L/min)** | **Ratio (gas:blood)** |
| --- | --- | --- |
| 100 | 1.5 | 15:1 |
| 200 | 3.0 | 15:1 |
| 300 | 4.5 | 15:1 |
| 400 | 6.0 | 15:1 |

System exchange, including replacement of the multiECCO_2_R membrane, was performed based on cumulative processed blood volume and device performance. The maximum cumulative blood volume per system is approximately 1250 L; **once this limit is reached, the extracorporeal system was exchanged, including replacement of the** multiECCO_2_R **filter, to ensure continued operation and optimal device performance**. The expected system runtime and exchange frequency depending on blood flow are summarized below.

| **Blood flow (mL/min)** | **Blood volume per day (L)** | **Time to 1250 L (days)** | **Expected system changes in 5 days** |
| --- | --- | --- | --- |
| 100 | 144 | ~8.7 | 0–1 |
| 200 | 288 | ~4.3 | 1–2 |
| 300 | 432 | ~2.9 | 2 |
| 400 | 576 | ~2.2 | 2–3 |

Calculation: Blood volume/day = blood flow × 60 × 24; Maximum cumulative volume per system ≈ 1250 L.

Over a treatment period of 5 days, this resulted in approximately 2–3 system exchanges, including multiECCO_2_R filter replacement, depending on the applied blood flow rate.

**CRRT dose and anticoagulation management**

CRRT was delivered with a prescribed effluent dose of 30–35 mL/kg/h, accounting for potential downtime to ensure adequate delivered dose.

Anticoagulation strategies differed between treatment groups according to the extracorporeal modality. In the standalone miECCO_2_R group, systemic anticoagulation was primarily performed using unfractionated heparin, titrated to a target activated partial thromboplastin time of 60–80 seconds; argatroban was used in cases of suspected or confirmed heparin-induced thrombocytopenia.

In the combined miECCO_2_R/CRRT group, CRRT was performed using regional citrate anticoagulation. A calcium-free dialysis solution and 4% trisodium citrate were used, targeting a post-filter ionized calcium concentration of 0.25–0.35 mmol/L. Systemic ionized calcium levels were regularly monitored and substituted as required according to institutional protocols.

**eTable 1. Primary indication for RRT initiation in the miECCO_2_R/CRRT subcohort (n = 9)**

| **Primary indication** | **Definition*** | **n (%)** |
| --- | --- | --- |
| AKI stage 3 (creatinine and urine output criteria) | Increase in serum creatinine ≥3× baseline and/or urine output <0.3 mL/kg/h for ≥24 h or anuria ≥12 h | 5 (55.6%) |
| AKI stage 3 (creatinine criterion only) | Increase in serum creatinine ≥3× baseline or ≥4.0 mg/dL | 2 (22.2%) |
| AKI stage 2 (creatinine criterion only) with additional indication | AKI stage 2 (creatinine 2.0–2.9× baseline) with **severe metabolic acidosis (pH <7.20)** | 2 (22.2%) |

AKI: acute kidney injury, KDIGO: Kidney Disease: Improving Global Outcomes, miECCO_2_R: minimally invasive extracorporeal carbon dioxide removal, miECCO_2_R/CRRT: minimally invasive extracorporeal carbon dioxide removal combined with continuous renal replacement therapy.

*Definitions are based on the 2012 KDIGO Clinical Practice Guideline for AKI (3).

**eTable 2. Characteristics of the patients in the study cohort (n = 20)**

| **Baseline characteristics** | **Value** |
| --- | --- |
| Age, years | 59.5 (53.5–67.0) |
| Female sex, n (%) | 9 (45%) |
| BMI, kg/m^2^ | 26.6 (24.0–30.0) |
| **Clinical scores** |  |
| SOFA | 12 (8–14) |
| SAPS II | 66 (50–70) |
| APACHE II | 28 (23–33) |
| **COVID-19 ARDS, n (%)** | 9 (45%) |
| **Non-COVID ARDS, n (%)** | 11 (55%) |
| **Ventilation, n (%)**   - Invasive - Non-invasive | 17 (85%)  3 (15%) |
| **Comorbidities, n (%)** |  |
| Arterial hypertension | 9 (45%) |
| Chronic kidney disease | 4 (20%) |
| Coronary artery disease   - LVEF <40% | 8 (40%)  3 (15%) |
| Diabetes mellitus | 10 (50%) |
| **Supportive therapies, n (%)** |  |
| Prone positioning | 9 (45%) |
| NO inhalation | 9 (45%) |
| Tracheostomy | 10 (50%) |
| Vasopressor support | 15 (75%) |
| **Therapy duration and mortality** |  |
| Duration of miECCO_2_R therapy, days | 7.5 (5.0–9.0) |
| Mortality during observation period (5 days), n (%) | 3 (15%) |
| 28-day mortality, n (%) | 9 (45%) |

*Abbreviations*: APACHE II: acute physiology and chronic health evaluation score, BMI: body mass index, COPD: chronic obstructive pulmonary disease, LVEF: left ventricular ejection fraction, NO: nitric oxide, SAPS II: simplified acute physiology score, SOFA: sequential organ failure assessment score.

Data are presented as median (25%–75% interquartile range) or absolute numbers (n) with percentage (%).

**eTable 3. Baseline characteristics and complications observed in the study population stratified by treatment subgroup (miECCO_2_R standalone vs. miECCO_2_R/CRRT)**

| **Characteristics/Parameter** | **miECCO_2_R**  **(n = 11)** | **miECCO_2_R/CRRT**  **(n = 9)** | **P-Value** |
| --- | --- | --- | --- |
| Age, years | 62 (55–66) | 57 (52–67) | 0.59 |
| Sex, male/female, n (%) | 7/4 (64%/36%) | 4/5 (45%/55%) | 0.65 |
| BMI, kg/m^2^   - Obesity (BMI > 30 kg/m²) | 26,1 (23.8–28.1)  3 (27%) | 27.5 (24.3–30.1)  4 (44%) | 0.21  0.64 |
| Duration of miECCO_2_R therapy, days | 8 (7–10) | 5 (3–8) | 0.22 |
| Type of ventilation:   - Invasive - Non-invasive | 8 (73%)  3 (27%) | 9 (100%)  0 (–) | 0.22  NE |
| 28-day mortality | 4 (37%) | 5 (56%) | 0.65 |
| Tracheostomy | 5 (45%) | 5 (56%) | 1.00 |
| Prone positioning | 3 (27%) | 6 (67%) | 0.17 |
| NO inhalation | 3 (27%) | 6 (67%) | 0.17 |
| Vasopressor support | 6 (55%) | 9 (100%) | 0.04 |
| COVID-19 ARDS | 3 (27%) | 6 (67%) | 0.17 |
| Heparin | 11 (100%) | 0 (–) | NE |
| Citrate | 0 (–) | 9 (100 %) | NE |
| SAPS II | 57 (42–66) | 67 (67–72) | 0.11 |
| SOFA | 10 (6–11) | 13 (12–15) | 0.04 |
| APACHE II | 24 (18–29) | 32 (27–34) | 0.10 |
| Membrane lung clotting | 1 (9%) | 3 (33%) | 0.28 |
| Thrombocytopenia* | 0 (–) | 1 (11%) | NE |

Note: continuous variables were compared using the Mann-Whitney U test, whereas categorical variables were analyzed using Fisher’s exact test; two-tailed P-values were reported. Data are presented as median (25%–75% interquartile range) or absolute numbers (n) with percentage (%).

*Thrombocytopenia was defined as a platelet count below 50.000 per microliter. One patient had a baseline platelet count of 52.000, and the thrombocytopenia observed during subsequent visits was considered unrelated to miECCO_2_R therapy.

APACHE II: acute physiology and chronic health evaluation II, ARDS: acute respiratory distress syndrome, BMI: body mass index, COVID-19: coronavirus disease 2019, miECCO_2_R: minimally invasive extracorporeal carbon dioxide removal, miECCO_2_R/CRRT: minimally invasive extracorporeal carbon dioxide removal combined with continuous renal replacement therapy, NE: not estimated, SAPS II: simplified acute physiology score II; SOFA: sequential organ failure assessment score.

**eTable4. Blood gas, ventilator, hemodynamic, and laboratory parameters at baseline and during miECCO_2_R therapy**

|  | **Baseline**  **(n = 20)** | **1 h**  **(n = 20)** | **4 h**  **(n = 20)** | **24 h**  **(n = 20)** | **2 d**  **(n = 20)** | **3 d**  **(n = 17)** | **5 d**  **(n = 15)** | **Friedman Test - χ^2^** | **P-Value** | **Skillings–Mack - χ^2^** | **P-Value** |
| --- | --- | --- | --- | --- | --- | --- | --- | --- | --- | --- | --- |
| **Blood gases** | | | | | | | | | | | |
| PaCO_2_, mm Hg | 71.4 (64.2–77.5) | 62.1 (50.1–67.8)** | 55.9 (47.5–63.9)*** | 51.6 (45.1–57.7)*** | 49 (45.4–54.1)*** | 49.5 (45.3–57.1)*** | 46.1 (43.8–64.2)*** | 41.08 | <0.001 | 55.64 | <0.001 |
| CO_2_ clearance, ml/min | NA | 82.9 (50.7–110.7) | 48.3 (31.6–97.6) | 58.5 (47.6–96.5) | 62.7 (42.6–73.7) | 81.8 (51.4–88.4) | 72.0 (47.5–89.4) | 9.69 | 0.0846 | 13.71 | 0.0175 |
| CO_2_, pre-post membrane, mm Hg | NA | 49.7 (42.2–56.2) | 49.6 (45.2–52.3) | 40.4 (35.5–47.4) | 40.2 (37.2–46.0) | 40.7 (36.2–45.9) | 37.6 (33.1–51.0) | 4.09 | 0.537 | 11.98 | 0.0351 |
| pH | 7.28 (7.21–7.36) | 7.32 (7.25–7.42) | 7.39 (7.31–7.43)** | 7.40 (7.36–7.46)*** | 7.39 (7.36–7.46)*** | 7.40 (7.37–7.43)*** | 7.39 (7.34–7.44)** | 37.85 | <0.001 | 45.38 | <0.001 |
| PaO_2_, mm Hg | 79 (69–88) | 70 (60–76) | 73 (60–79) | 77 (71–80) | 70 (65–81) | 76 (70–79) | 75 (68–81) | 8.05 | 0.234 | 9.94 | 0.1271 |
| Bicarbonate, mmol/L | 33.1 (28.4–35.5) | 31.4 (26.6–34.8) | 31.9 (27.5–34.6) | 31.4 (28.2–33.2) | 30.8 (27.4–34.4) | 29.2 (27.6–33.8) | 29.7 (27.5–32.5) | 10.76 | 0.096 | 9.77 | 0.1349 |
| **Ventilator parameters** | | | | | | | | | | | |
| Driving pressure, cm H_2_O | 22 (19–22) | 20 (17–22) | 19 (17–21) | 20 (17–22) | 18 (15–22) | 17 (15–20)* | 15 (13–18)** | 31.88 | <0.001 | 27.97 | <0.001 |
| PInsp, cm H_2_O | 30 (27–32) | 28 (26–30) | 27 (26–30) | 28 (25–30) | 28 (25–30) | 26 (24–27)** | 25 (19–27)** | 38.28 | <0.001 | 27.68 | <0.001 |
| PEEP, cm H_2_O | 8 (6–10) | 8 (6–10) | 8 (6–10) | 8 (6–10) | 8 (6–10) | 7 (6–10) | 7 (6–9) | 4.69 | 0.583 | 1.96 | 0.923 |
| Tidal volume, ml/kg | 6.1 (4.4–7.6) | 5.7 (4.1–7.4) | 5.4 (4.1–7.3) | 5.6 (4.0–6.7) | 4.8 (3.7–7.1) | 6.2 (4.2–7.2) | 6.2 (5.1–7.2) | 5.62 | 0.467 | 9.47 | 0.1486 |
| FiO_2_, % | 48 (35–61) | 50 (40–65) | 50 (40–66) | 45 (35–56) | 45 (35–56) | 45 (40–50) | 40 (30–50) | 13.87 | 0.031 | 11.26 | 0.081 |
| PaO_2_/FiO_2_ | 158.0 (127.1–221.1) | 132.5 (109.3–167.5) | 159.9 (102.5–195.6) | 146.9 (125.6–210.6) | 161.5 (124.8–201.5) | 165.6 (134.2–191.9) | 185.0 (148.9–252.5) | 15.72 | 0.015 | 18.49 | 0.005 |
| Respiratory rate, breaths/min | 21 (18–30) | 20 (19–30) | 20 (19–26) | 22 (16-26) | 22 (15–28) | 20 (17–25) | 25 (16–28) | 5.86 | 0.439 | 3.41 | 0.755 |
| Dynamic respiratory compliance, mL/cmH_2_O | 17.16 (11.00–29.85) | 16.09 (11.41–29.11) | 16.41 (11.47–29.16) | 17.33 (12.11–23.62) | 16.07 (11.57–24.28) | 22.94 (13.00–34.71) | 28.42 (13.10–35.65) | 14.96 | 0.021 | 14.67 | 0.023 |
| Ventilatory ratio | 2.74 (2.61–3.02) | 2.11 (1.87–2.42)*** | 1.88 (1.78–2.41)*** | 1.80 (1.52–2.22)*** | 1.83 (1.19–2.15)*** | 1.88 (1.56–1.96)*** | 1.92 (1.33–2.26)*** | 40.68 | <0.001 | 51.23 | <0.001 |
| Mechanical power, J/min | 31.93 (21.39–41.59) | 29.45 (18.66–35.06) | 31.65 (17.22–36.25) | 27.20 (15.60–33.91) | 20.53 (18.35–30.52) | 24.65 (16.39–30.36) | 19.99 (14.71–44.64) | 11.24 | 0.081 | 12.80 | 0.046 |
| **Hemodynamics and vasopressor support** | | | | | | | | | | | |
| MAP, mm Hg | 76 (69–85) | 79 (69–93) | 76 (68–78) | 72 (63–81) | 76 (68–83) | 70 (67–77) | 71 (66–78) | 12.94 | 0.044 | 8.60 | 0.197 |
| Norepinephrine, µg/kg/min | 0.071 (0.001–0.25) | 0.114 (0.001–0.227) | 0.136 (0.028–0.18) | 0.04 (0.001–0.271) | 0.028 (0–0.171) | 0.031 (0–0.11) | 0.012 (0.001–0.062) | 18.99 | 0.004 | 8.93 | 0.178 |
| **Laboratory parameters** | | | | | | | | | | | |
| White blood cell count, g/L | 14.4 (9.5–21.1) | NE | NE | 14.2 (11.3–18.0) | 15.2 (11.3–17.6) | 13.8 (9.2–15.9) | 14.4 (11.6–17.2) | 11.81 | 0.019 | 7.84 | 0.098 |
| Hemoglobin, g/dL | 96 (90–112) | NE | NE | 92 (83–106) | 91 (84–105) | 86 (80–95)* | 85 (83–96) | 22.05 | <0.001 | 20.72 | <0.001 |
| Hematocrit,  L/L | 0.30 (0.29–0.35) | NE | NE | 0.28 (0.26–0.32) | 0.29 (0.27–0.32) | 0.28 (0.24–0.29)* | 0.27 (0.26–0.30) | 30.70 | <0.001 | 29.42 | <0.001 |
| Platelet count, giga/L | 263 (211–344) | NE | NE | 246 (119–286) | 208 (149–269) | 198 (116–256)* | 144 (120–260)* | 20.29 | <0.001 | 24.75 | <0.001 |
| **Serum creatinine, mg/dL** | 1.3 (0.8–2.0) | NE | NE | 1.0 (0.8–1.4) | 0.8 (0.7–1.0) | 0.8 (0.6–1.0) | 0.9 (0.6–1.2) | 4.08 | 0.396 | 6.11 | 0.296 |
| **Urea, mg/dL** | 86 (54–195) | NE | NE | 90 (62–112) | 58 (41–109) | 62 (38–105) | 69 (32–112) | 4.18 | 0.382 | 5.06 | 0.409 |
| Lactate dehydrogenase, U/L | 327 (236– 570) | NE | NE | 358 (275–530) | 366 (274– 528) | 334 (287– 536) | 350 (322– 474) | 0,32 | 0,99 | 2.00 | 0.849 |
| Haptoglobin, g/L^#^ | 2.36 (1.01–3.27)  N=9 | NE | NE | 1.31 (0.67–2.16) N=4 | 2.32 (1.71–2.96) N=5 | 1.36 (0.91–1.81) N=3 | 1.32 (1.18–1.77) N=9 | NE | NE | NE | NE |

*p < 0.05, **p < 0.01, ***p < 0.001 in comparison with baseline values, as determined by post-hoc analysis.

^#^Statistical analysis was not performed for haptoglobin because it was not routinely measured in the study, and the number of observations varied between visits (n = 3–9).

NA: not applicable, NE: not estimated, MAP: mean arterial pressure, PInsp: peak inspiratory pressure, PEEP: positive end-expiratory pressure, PaO_2_: partial pressure of arterial oxygen, FiO_2_: fraction of inspired oxygen. Dynamic respiratory compliance (4), mechanical power (5, 6), ventilatory ratio (7) and CO_2_ clearance (8, 9) were calculated as previously described in the corresponding literature.

**eTable5. Ventilator parameters at baseline and during miECCO_2_R therapy observed in the study population stratified by treatment subgroup (miECCO_2_R standalone vs. miECCO_2_R/CRRT)**

|  | **Baseline** | | **1 h** | | **4 h** | | **24 h** | | **2 d** | | **3 d** | | **5 d** | |
| --- | --- | --- | --- | --- | --- | --- | --- | --- | --- | --- | --- | --- | --- | --- |
|  | **miECCO_2_R**  **(n=11)** | **miECCO_2_R/**  **CRRT (n=9)** | **miECCO_2_R**  **(n=11)** | **miECCO_2_R/**  **CRRT (n=9)** | **miECCO_2_R**  **(n=11)** | **miECCO_2_R/**  **CRRT (n=9)** | **miECCO_2_R**  **(n=11)** | **miECCO_2_R/**  **CRRT (n=9)** | **miECCO_2_R**  **(n=11)** | **miECCO_2_R/**  **CRRT (n=9)** | **miECCO_2_R**  **(n=10)** | **miECCO_2_R/**  **CRRT (n=7)** | **miECCO_2_R**  **(n=10)** | **miECCO_2_R/**  **CRRT (n=5)** |
| Driving pressure, cm H_2_O | 22  (20–24) | 22  (16–22) | 20  (19–24) | 17  (16–22) | 20  (18–22) | 17  (16–19) | 20  (18–23) | 19  (16–22) | 20  (16–22) | 17  (15–20) | 18  (15–20) | 16  (16–18) | 16  (13–19) | 15  (14–16) |
| PInsp, cm H_2_O | 30  (27–32) | 29  (27–30) | 29  (26–32) | 27  (25–30) | 27  (26–31) | 27  (25–29) | 27  (25–30) | 28  (25–30) | 27  (24–30) | 28  (25–30) | 26  (22–29) | 26  (26–26) | 24  (20–27) | 25  (18–27) |
| PEEP, cm H_2_O | 8  (6–10) | 8 (7–11) | 7  (6–10) | 10 (7–10) | 7  (6–10) | 10  (7–10) | 6  (6–8) | 10  (7–10) | 7  (6–8) | 10  (7–10) | 7  (6–9) | 9  (6–10) | 7  (6–8) | 6  (6–10) |
| Tidal volume, ml/kg | 6.4  (5.2–7.5) | 4.8  (4.3–7.6) | 6.4  (5.1–7.6) | 4.5  (3.9–5.6) | 6.4  (4.8–7.7) | 4.1  (3.5–5.2) | 6.2  (4.6–6.7) | 5.5  (3.6–6.6) | 4.8  (4.3–7.6) | 4  (3.7–5.5) | 6.8  (4.7–7.5) | 4.6  (4.2–5.8) | 6.4  (5.3–7.7) | 5.2  (3.7–6.6) |
| Respiratory rate,  breaths/min | 20  (16–30) | 23  (19–30) | 20  (15–27) | 21  (20–30) | 19  (16–22) | 23  (21–30) | 19  (15–25) | 23  (21–28) | 16  (14–25) | 23  (21–30) | 18  (15–27) | 22  (20–24) | 20  (15–27) | 25  (24–30) |
| PaO_2_/  FiO_2_ | 171.4 (154.5–224.8) | 145.5 (104.6–197.1) | 141.2 (120–185.5) | 115.4 (105.7–157.8) | 174 (118.5–197.5) | 137.3 (91.1–165.8) | 170 (131–222.1) | 133.4 (108.8–153.4) | 170.2 (159.8–201.5) | 130.9 (115.8–162.9) | 165.6 (133.6–187.5) | 173.2 (148.2–194.4) | 223.8 (149.3–252.5) | 149.4 (146.7–219.5) |

No statistical differences were observed between the ventilator parameters at any time-point throughout the study visits when comparing values of the miECCO_2_R and miECCO_2_R/CRRT groups; thus, no p-values are displayed.

PInsp: peak inspiratory pressure, PEEP: positive end-expiratory pressure, PaO_2_: partial pressure of arterial oxygen, FiO_2_: fraction of inspired oxygen.

**References**

1. Eloot S, Peperstraete H, De Somer F, Hoste E. Assessment of the optimal operating parameters during extracorporeal CO2 removal with the Abylcap(R) system. Int J Artif Organs. 2017;39(11):580-5.

2. Hermann A, Riss K, Schellongowski P, Bojic A, Wohlfarth P, Robak O, et al. A novel pump-driven veno-venous gas exchange system during extracorporeal CO2-removal. Intensive Care Med. 2015;41(10):1773-80.

3. Kidney Disease: Improving Global Outcomes (KDIGO) Acute Kidney Injury Work Group. KDIGO Clinical Practice Guideline for Acute Kidney Injury. Kidney international.Suppl. 2012(2):1–138.

4. Tawfik P, Syed MKH, Elmufdi FS, Evans MD, Dries DJ, Marini JJ. Static and Dynamic Measurements of Compliance and Driving Pressure: A Pilot Study. Front Physiol. 2022;13:773010.

5. Chiumello D, Gotti M, Guanziroli M, Formenti P, Umbrello M, Pasticci I, et al. Bedside calculation of mechanical power during volume- and pressure-controlled mechanical ventilation. Crit Care. 2020;24(1):417.

6. Santarisi A, Suleiman A, Talmor DS, Goodspeed V, Schaefer MS, Baedorf Kassis EN. Simplified Mechanical Power Calculation in Patients Receiving Pressure-Regulated, Volume-Targeted Hybrid Modes of Ventilation. Respir Care. 2024;69(3):349-53.

7. Sinha P, Fauvel NJ, Singh P, Soni N. Analysis of ventilatory ratio as a novel method to monitor ventilatory adequacy at the bedside. Crit Care. 2013;17(1):R34.

8. May AG, Sen A, Cove ME, Kellum JA, Federspiel WJ. Extracorporeal CO(2) removal by hemodialysis: in vitro model and feasibility. Intensive Care Med Exp. 2017;5(1):20.

9. Allescher J, Rasch S, Wiessner JR, Perez Ruiz de Garibay A, Huberle C, Hesse F, et al. Extracorporeal carbon dioxide removal with the Advanced Organ Support system in critically ill COVID-19 patients. Artif Organs. 2021;45(12):1522-32.
